# 

**Published:** 1894-07

**Authors:** 


### Society Meetings.

The Medical Society of the County of Chautauqua will hold its annual meeting at the Thompson House, Mayville, and the Hotel Atheneum, Chautauqua, on Tuesday, July 10, 1894, under the presidency of Dr. Nelson G. Richmond, of Fredonia. The scientific program includes a discussion on diphtheria, in several prepared papers and a number of appointed referees. Dr. C. A. Ellis, of Sherman, is secretary, and deserves great credit for the preparation of such an interesting program. The society is rapidly going to the front among the very best county medical societies in the State.

The Medical Society of the County of Cattaraugus held its annual meeting at Little Valley on Thursday, May 24, 1894. The following officers were elected: President, Dr. W. B. Johnson, Ellicottville; vice-president, Dr. Edward Torrey, Allegany; secretary and treasurer, Dr. M. C. Hawley, East Randolph; censors, Dr. J. E. K. Morris, Olean; Dr. F. C. Beals, Salamanca; Dr. E. M. Shaffner, Great Valley; committee on program, Dr. Edward Torrey, Allegany; Dr. J. C. Clark, Olean; Dr. L. L. Deck, Salamanca.

The International Congress of Obstetrics and Gynecology will hold its second session at Geneva, Switzerland, in September, 1896. The questions for discussion are: (1) The Treatment of Eclampsia; (2) Surgical Treatment of Uterine Retrodeviations; (3) Relative frequency of different kinds of Narrowing of the Pelvis in different countries; (4) The Best Way of Suturing the Abdominal Parietes in order to avoid Eventration; (5) Treatment of Pelvic Suppurations.

The American Academy of Medicine will hold its next annual meeting at Jefferson, N. H., August 29 and 30, 1894. This society has acted wisely in severing itself from the American Medical Association, to which it has served as an appendage quite long enough.

### Hospital Notes.

### Academy of Medicine Notes.

### Personal.

Dr. Henry C. Buswell, of Buffalo, who has spent the past year in Europe, will delay his return to the United States until Autumn. At present he is serving as resident physician in one of the large Vienna hospitals, that affords him a rare opportunity for the prosecution of the special work for which he is preparing himself. We shall welcome Dr. Buswell's return with pleasure.

### Society Meetings.

The Association of Erie Railway Surgeons, at its meeting to be held at the International hotel, Niagara Falls, July 18, 1894, offers the following program: The Peritoneum from a Surgical Standpoint, by Prof. J. B. Murphy, M. D., Chicago, Ill.; discussion opened by Prof. Roswell Park, M. D., Buffalo, N. Y. Medical Expert Testimony, by Prof. Wm. B. Outten, M. D., chief surgeon M. P. R. R., ex-president National Association Railroad Surgeons, St. Louis, Mo.; discussion opened by Prof. J. B. Murdock, M. D., Pittsburg, Pa. Delayed Union and Pseudo Arthrosis, by Wm. H. Buechner, M. D., Youngstown, O.; discussion opened by C. M. Daniels, M. D., ex-president of Association Erie R. R. Surgeons. Subject to be announced, by R. Sayer Harnden, M. D., ex-president Association of Erie Railway Surgeons. Association Work of the Railway Surgeon—Its Object, Scope, Benefits and Limits, by S. Birdsall, M. D., Susquehanna, Pa. Tetanus, by Emery H. Leyman, M. D., Huntington, Ind. Reminiscences of a Railroad Collision, by A. G. Ellinwood, M. D., Attica, N. Y. Conservatism in Railroad Surgery, by F. W. Thomas, M. D., Marion, O. Is Alcohol a Stimulant, a Food or a Sustainer of Vitality? by W. V. R. Blighton, M. D., Tonawanda, N. Y. The Essentials of Asepsis, by J. S. Mudge, M. D., Olean, N. Y. Officers for 1894—President, C. B. Kibler, M. D., Corry, Pa.; vice-president, E. Griswold, M. D.,

Sharon, Pa.; secretary and treasurer, W. W. Appley, M. D., Cochecton, N. Y. Committee—C. M. Daniels, Buffalo, N. Y.; J. L. Eddy, M. D., Olean, N. Y.; C. B. Kibler, M. D., Corry, Pa.

### Literary Notes.

MERRITT H. CASH PRIZE FUND.—For the information of all concerned, I desire to state that the Medical Society of the State of New York offers a prize of $100, payable from the Merritt H. Cash Prize fund, for the best original essay on any medical or surgical subject.

The conditions are: That the competitor shall reside in the State of New York and shall be a member of a county medical society; that the essay shall be either printed or type-written; that each essay shall be designated by a motto on the title page, and accompanied by a sealed paper bearing the same motto and enclosing the name of its author, in order that the name of the successful author alone may be ascertained; and that all essays shall be sent to the chairman of the committee on prize essays prior to January 1st next.

The committee on prize essays are: Dr. Franklin Townsend, Jr., 2 Park Place, Albany; Dr. A. Walter Suiter, Herkimer; Dr. Charles Stover, Amsterdam.

Secretaries of county medical societies are requested to communicate this to the members of their societies.

F. C. CURTIS, *Secretary.*

THE *Archives of Pediatrics* will be edited by Dillon Brown, M. D., adjunct professor of pediatrics at the New York Polyclinic, commencing with the July issue, 1894. Exchanges should be addressed, Editor *Archives of Pediatrics*, 40 East 57th street, New York.

MESSRS. F. B. VANDEGRIFT & Co., of 50 South Fourth street, Philadelphia, and 27 William street, New York, are making a digest of the tariff bill that the present congress is considering, which they propose to publish within seventy-two hours after the President shall have signed it. This will be a novelty in the way of tariff publications, and gives the list of articles classified under their proper headings for ready reference, together with the rate of duty, paragraph of the law and decisions of the courts ; also the allowance of wastage, showing duty to be returned on manufactured articles exported with benefit of drawback, the value of all foreign coins, a condensed express tariff, and other useful matters in connection with the customs service.

---

THE *Kansas Medical Journal* has appeared as a weekly during the past six months. It seemed a dangerous experiment to change from a monthly to a weekly, especially when we consider the difficulties that surround medical journalism at the present time. But our Kansas contemporary apparently has scored a conspicuous success and deserves congratulations thereupon.

---

THE *Medical Mirror*, of St. Louis, has appeared in a handsome new dress. Such an evidence of prosperity, as is indicated by the June issue of the *Mirror*, is pleasant to behold, and we congratulate the editor, who can look into his mirror with pride.

---

THE *Railway Surgeon* made its first appearance under the date of June 5, 1894. It is the organ of the National Association of Railway Surgeons, taking the place of the *Railway Age*.

---

IN ANNOUNCING the completion of An American Text-Book of Practice, the publisher, Mr. William B. Saunders, asserts that in this work over 500 pages are from the pen of Dr. William Pepper. This fact, from a purely mechanical standpoint in these days of enormous literary production, would not appear in itself to be matter for special comment, but when there is taken into account the editor's busy life, it is a notable instance of the wonderful vitality and executive ability of an exceptionally gifted man.

---

DR. WILLIAM M. MCLAURY, of New York, furnishes the description of an oldtime medical work that will interest antiquarians and medical men generally. It has been presented to the New York Academy of Medicine and may be found in the library there. The following is a brief description of the book :

The History of the Absorbent System, containing the chylography or description of the human lacteal vessels, with the different methods of discovering, injecting and repairing them, and the instruments used for these purposes.

Illustrated by "figures," by John Sheldon, surgeon, F. R. S., professor of anatomy, physiology, etc., etc., and surgery. London. 1784.

This old volume has the name of D. Hosack written on the title page. It is dedicated to Sir Joseph Bank, Bart., president of the Royal Society, etc., etc.

Then comes a long list as subscribers. Among them are: Robert Adair, Esq., surgeon to Chelsea Hospital and inspector general to the army; Sir George Baker, Bart., F. R. S., physician to Her Majesty; William Broomfield, Esq., surgeon to Her Majesty's household; Richard Budd, M. D., physician to St. Bartholomew's Hospital; John Beich, surgeon extraordinary to His Royal Highness, the Prince of Wales; Denzil Bragge, surgeon, Axminster, Devon.; Mr. John Crawford, surgeon, Barbadoes; William Denman, M. D., teacher of midwifery; J. S. Haufman, M. D., professor of anatomy, etc., etc.; Mr. John Harris, army surgeon; Mr. George Hughes, navy surgeon; Mr. John Jaffrey, navy surgeon; Mr. John Neale, Nottingham; Antonio Sehapa, M. D., James Simms, M. D.; Mr. Josiah Teed, surgeon; Henry Watson, Esq., F. R. S., Mr. William Wheatley; Thomas Webb, surgeon.

This quaint, old book has written on the fly leaf, "To Mr. Harwood, with the author's compliments."

---

The *Rocky Mountain Globe* is a handsomely illustrated paper, giving photographic reproductions and etchings of the picturesque scenery with which the Rockies abound. It is published at Denver, Colorado, and its subscription price is $1 a year. The issue of April 1, 1894, is a handsome specimen of press and newspaper work.

---

Teratologia is the title of a magazine to contain quarterly contributions to antenatal pathology with reviews of the current literature of the subject. It is edited by J. W. Ballantyne, M. D., and published by Williams & Norgate, London and Edinburgh. Dr. Ballantyne, 24 Melville street, Edinburgh, will be glad to receive any books or other publications bearing on teratology, for review in this periodical.

---

The *Bristol Medico-Chirurgical Journal* has lately come to our exchange table. It is a quarterly, published under the auspices of the Bristol Medico-Chirurgical Society, and edited by R. Shingleton Smith, M. D., and published by J. W. Arrowsmith, Bristol.

It is a well-printed magazine, containing seventy-eight pages, and in the number for March is published an interesting article on Appendicitis, by Dr. James Swain, and another on Intussusception, by Mr. Arthur W. Prichard.

---

THE *Maryland Medical Journal* has just put on a new dress and appears in new form. It has, indeed, undergone a complete rehabilitation, and is now one of the handsomest medical weeklies that comes to our exchange table.

---

NOW READY. — A Biography of Eminent American Physicians and Surgeons, edited by R. French Stone, M. D., author of Elements of Modern Medicine, surgeon general National Guard, State of Indiana, consulting physician to the Indianapolis City Hospital and Dispensary, ex-president of the Marion County Medical Society, member of the Indiana State Medical Society and American Medical Association, formerly professor of materia medica, therapeutics and clinical medicine in the Central College of Physicians and Surgeons, etc. Illustrated with hundreds of fine photo-engraved portraits and autographs. Published by Carlon & Hollenbeck, of Indianapolis, in one large octavo volume, containing 751 double-columned printed pages, and to be sold by subscription only. Price, in cloth, $8 ; leather, $9, and half morocco, $10, sent C. O. D., express charges prepaid. Orders will be received by Dr. Stone, editor and business manager, 16 West Ohio street, Indianapolis, and by authorized state agents.

---

FIRST, GET A MAN.—When I asked our wise doctor at home what sort of a physician I should choose in the West (meaning to what school he should belong, since some schools are so much better represented than others in the West), he answered briefly, as if the question made him " tired : " " First, get a man," writes that most charming writer, Mary Hallock Foote, in reply to the question, " What Constitutes a Good Husband ?" in the July *Ladies' Home Journal.*

So I think we mothers might say to the girls, if it were at all supposable that any girl would ever ask the question, what are the best masculine qualities conducive to a wife's happiness : " First, get a man." Manliness in the highest sense of the word is surely the natural, and, therefore, must be the lasting bond in marriage.

### Index to Volume XXXIII.

PAGE.

ABDOMINAL SURGERY in North Germany . . . . . . 673
Abscess, Sub-occipital . . . . . . 337
Academy of Medicine Notes . . . . 52, 116, 161, 238, 303, 377, 695, 750
Acid, Acetic, as a Germicide . . . 73
Address, Annual, President Medical Society of the County of Erie 449
Aldehyde, Formic, as an Ocular Antiseptic . . . . . . . . . . . 348
Almshouse, Cases from the Medical Service at the Cattaraugus County . . . . . . . . . . . . . . . 336
Ambulance Car for Street Railways . . . . . . . . . . . . . 234
American Medical Editors' Association Banquet . . . . . . . . . . 169
Amputation, Double Synchronous of Legs . . . . . . . . . . 74
Anemia, Causation of and Blood Changes Produced by Uric Acid. 158
Angell, Edward B., M.D. Double Lesion of the Brain—Cerebral Cyst—Cerebellar Tumor . . . . 538
Angina Pectoris, Nature and Treatment of . . . . . . . . . . . 351
Angio-neurotic Edema, Case of . . 286
Arteries, Torsion and the Homologous Ligation of Divided . . . . 257
Army Medical Board . . . . . 448, 488
Medical School . . . . . 231
Medical Corps, Examinations of . . . . . . . . . 573
Aphasia, Motor following Apoplexy 340
Asphyxia, Intrauterine . . . . . . 65
Neonatorum, New Method of Establishing Artificial Respiration in . . . . . 665
Aural Reflex, Due to Impacted Wax 351

BARR, A. D., M. D. Physiology of Conception . . . . . . 605
Bartlett, F. W., M. D. Gift of to the Buffalo Academy of Medicine . . . . . . . 298
How shall we Treat Scarlet Fever? . . . . . . . . . 78
Beriberi, Outbreaks of on Ships . . 99
Bladder Gymnastics and Auto-irrigation . . . . . . . . . . 714
Blepharitis Marginalis, Hydrogen Dioxid in . . . . . . . . . . . 354

PAGE.

Bloodless Amputation of the Hip—Lanphear . . . . . . . . . . . . 89
Board of State Medical Examiners . 744
Books received . . . . 60, 125, 187, 254 317, 376, 444, 509, 571, 638, 702, 762
Bowlegs . . . . . . . . . . . . . . 109
Brain, Double Lesion of . . . . . 538
Breech Presentations . . . . . . . 738
Buffalo Medical College Professors and Reduction of State Licensing Fees . . . . . . 618
Medical and Surgical Journal, Compliment to . . . 741
Burr, C. B., M. D. Paranoia, with Delusions of Change in Sex . . . 193

CARDIAC AFFECTIONS, Rest in . . . . . . . . . . . . . . 543
Car, A Hospital on Central Railroad of New Jersey . . . . . 617
Cars, Special for Phthisical Patients 550
Cary, Charles, M. D. Cause of Typhoid Fever in General and of the Epidemic in Buffalo in Particular . . . . . . . . . . . . . 641
Cataract, Lectures on at the Philadelphia Polyclinic . . . . . . . 232
Cerebral Surgery, Scope and Limitations of . . . . . . . . . . . 108
Charcot, Life and Work of . . . . 582
Cholecyst-duodenostomy and Gastro-enterostomies by aid of Murphy's Button . . . . . . . . . . 718
Chorea, Hereditary . . . . . . . 337
Christian Science, Attempt to Legalize . . . . . . . . . . . . . . 553
Church Bell Nuisance . . . . . . 363
Clark, Edward, M. D. Causes and Modes of Communication of Contagious and Infectious Diseases . . . . . . 201
Horace, M. D. Laryngeal Tuberculosis, a Typical Case with Illustration . . 275
Clausius, M. F., M. D. Translation by—Late Researches in Epilepsy . . . . . . . . . . . . . . 596
Clinical Memoranda, from the Sisters of Charity Hospital . . . . . . 221, 281, 341, 415, 532, 588, 718
Civil Service Examination . . . . . . . . . . . . . . 53, 255, 512, 639

PAGE.

College of Physicians and Surgeons of Ontario . . . . . . . . . . . . 231
Colorado Medical Library Association . . . . . . . . . . . . . . . 232
Comstock, Anthony, and the Daggett Table Company . . . . . . 299
Conception, Physiology of . . . . 605
Constitution Convention and Medical Interests . . . . . . . . . 742
Consumption, a Communicable Disease . . . . . . . . . 296
  And Travel . . . . . . . . 360
  The Prevention of . . . . . 648
Cordier, A. H., M. D., Surgical Problems in Intra-pelvic and Abdominal Diseases . . . . . . . 727
Cornea, Ulcers and Abscesses of . . 348

**Correspondence :**

  Letter from Frank W. Abbott, M. D. . . . . . . . . . 545
  Mr. Lawson Tait . . . . 424
Cott, George F., M. D. Intubation 137
County Medical Societies, Importance of Membership in . . . . . 690
Craig Colony for Epileptics . . . . 688
Crockett, M. A., M. D. Observations on Results of Removal of Diseased Uterine Appendages . . 385
Cushing, Clinton, M. D. Abdominal Surgery in North Germany . 673

DAGGETT, B. H., M. D. Concerning Posture . . . . . 85
  Speech of at Niagara Alumni Association Banquet . . . 684
  Bladder Gymnastics and Auto-irrigation . . . . . . 714
Daggett Table Co. and Mr. Comstock . . . . . . . . . . . . . . 385
Death, Sudden, from Unknown Cause . . . . . . . . . . . . . . 339
Dew, J. Harvie, M. D. New Method of Establishing Artificial Respiration in Asphyxia Neonatorum . . . . . . . . . . . . . 665
Diphtheria, Prevention of . . . . . 653
Disease, The Prevention of . . . . 1
  The Prevention of, a Problem for all Physicians . . 647
Diseases, Summer . . . . . . . . . 105
Doyle, Gregory, M. D. Double Synchronous Amputation of Legs in an Infant—Recovery . . . . . . . . . 74
  Fractures and their Treatment . . . . . . . . 465
Dysentery, Tannin and Boric Acid in . . . . . . . . . . . . . . 481

PAGE.

Dysmenorrhea . . . . . . . . . . . 487
Dyspepsia, Functional, So-called . 602

EAR COUGH . . . . . . . . . 106
  Edson, Cyrus, M. D., Evils of Substitution . . . . . . 287
Elbow-joint, Fractures at . . . . . 341
  Tuberculous Arthroitis of . . 285

**Editorials :**

  Buffalo University Medical College . . . . . . . . . . . . . 679
  College Commencements and Alumni Association Meetings 679
  First Pan-American Medical Congress . . . . . . . . . . 162
  Foghorn Nuisance . . . . . . 615
  Football in Colleges . . . . . 355
  Medical Education, Advanced 111
    Society of the State of New York . . . . . . . 484
  National Conference of State Medical Examining and Licensing Boards . . . 616
    National Medical Societies 739
  Need for Uniformity in Division of Abdominal Regions . 113
  Niagara University Medical College . . . . . . . . . . . 682
  Object Lesson in Hygiene . . 546
  Relation of Noises to Health Again . . . . . . . . . . . . 164
  Resignation of Dr. Rochester . 230
  Spelling of Medical Words . . 47
  Southern Surgical and Gynecological Association . . . . . 292
  State Examination for License. 227
    Examination and Public Health . . . . . . . . . 613
  Symphyseotomy . . . . . . . 430
Empyemia . . . . . . . . . . . . . 321
Epididymitis, Tuberculous . . . . 588
Epilepsy, Late Researches in, Translation . . . . . . . . . . . . . . 596
Epileptics, Craig Colony for . . . 688
Ethics, Medical, Revision of the Code of . . . . . . . . . 690
Examiners, North Carolina Board of Medical . . . . . . . . . . 549
Exercise, Some Reasons for Daily . 380

FEMUR, Acute Osteitis of Internal Condyle of . . . . . . . 282
Fibroids, Uterine . . . . . . . . 75
Fibroma, Molluscum . . . . . . . 338
Fog Horn Epidemic . . . . . . . 360
Fractures and Their Treatment . . 465

PAGE.

Fritz, William C., M. D. Case of Angio-neurotic Edema showing Remarkable Heredity . . . . . . 286

GONORRHEA, the Microscope in Examinations for . . . . 471
Gonorrheal Infection in Women . 654
Gould, George M., M. D. Spelling of Some Medical Words . . . 42
Gram, Franklin C., M. D. Statistics of Infectious Diseases . . . 197
Grand Army of the Republic . . . 235

HAMMOND, FREDERICK P., M. D. Superficial Laceration of Female Perineum . . . . . . . . . . . 157
Hart, Mr. Ernest. Address of at Pan-American Medical Congress . . . . . . 168
The Way He Registers . . . 169
vs. Wm. A. Hammond, M.D. 233
The Medical Mirror, The Post-Graduate and The Ohio Medical Journal . . 300
Hartwig, Marcell, M. D. Prostatic Diseases of Old Age . . . . 397
Harvard Medical Alumni Association Bulletin . . . . . . . . . . . 235
Haynes, Francis L., M. D. White's Operation for Enlarged Prostate . 481
Health Circular. By Dr. E. Wende 320
Hegar's Sign of Pregnancy, Value of . . . . . . . . . . . . . . . . 706
Hemoptysis, Treatment of . . . . 160
Hernia, Operative Treatment for Radical Cure of Inguinal . . . . 146
Hip Splint with Traction Mechanism . . . . . . . . . . . . . . . 297
Himmelsbach, G. A., M. D. Translation by . . . . . . . . . . 471
Hopkins, Henry R., M. D. Artificial Immunity . . . . . . 527
James L., Esq. Some Suggestions on Life Insurance 609
Hospital Car, on Central Railroad of New Jersey . . . . . . 617
Physicians and the Courts . 434

**Hospital Notes:**

Brigham Hall . . . . . . . . . 488
Buffalo General Hospital . . . 694
Children's Hospital of Buffalo . 551
Cincinnati Hospital . . . . . 690
Emergency Hospital at the World's Fair . . . . . . . . 297
Erie County Hospital . . 236, 364

PAGE.

Fitch Hospital . . . . . . . . 694
Jennie Casseday Infirmary for Women . . . 48, 434, 743
Maryland Hospital for the Insane . . . . . . . . . . . . . 391
Rhode Island Hospital . . . . 491
Sheppard Asylum for Mental Diseases . . . . . . . . . . . 621
Sisters of Charity Hospital . . 694
Woman's Hospital and Foundling's Home, Detroit . . . . 750
Hoyt, Dr. Charles S. Report on Immigration by . . . . . . . . 689
Hulbert, George F., M. D. Intrauterine Asphyxia . . . . . . . . . 65
Humerus, Tuberculous Osteomyelitis of . . . . . . . . . . 283
Osteo-arthroitis of . . . . . 284
Hydronephrosis, Intermittent . . . 221
Hysterectomy, Disputed Points in . 26
For Fibrous Tumor Complicating Pregnancy . . . . . 619

ICE WATER, How to Keep During the Night . . . . . . . . 350
Illinois State Board of Health and Requirements for Admission to Medical Colleges . . . . . . . . 623

**Illustrations:**

A New Method of Artificial Respiration in Asphyxia Neonatorum. J. Harvie Dew, M. D. Fig. 1. Opening of the Epiglottis by Gravity . . 666
Fig 2. Depression of the Pelvis and Lower Extremities . 667
Fig. 3. Forcible Expiration by arching the lumbar region backward and bending the child upon itself . . . . . . 668
Fig. 4. Expulsion of Mucus by elevating buttocks and depressing head . . . . . 672
Bladder Gynastics and Auto-Irrigation. B. H. Daggett, M. D. Fig. 1. The Daggett Canula . . . . . . . . 714
Fig. 2. Posture for Auto-irrigation of the Bladder . . 715
Concerning Posture. B. H. Daggett, M. D. Fig. 1. Dorsal Posture, knees and thighs flexed . . . . . . . . . . . 86
Fig. 2. Knee-thigh-chest Posture . . . . . . . . . . 88
Hegar's Sign of Pregnancy. J. W. Long, M. D. Fig. 1.

PAGE.

Intra-vaginal Finger in Anterior Vaginal Fornix—Abdominal Hand behind Fundus . . . . . . . . . . . . 706
Fig. 2. Intra-vaginal Finger behind Cervix, Abdominal Hand between Symphysis and Fundus . . . . . . . . 707
Fig. 3. Hand between Symphysis and Fundus, Finger in Rectum . . . . . . . . . 707
Hydro-nephrosis from Valvular Stricture of the Ureter. Herman Mynter, M. D. Fig. 1. Incision through Valvular Stricture and Sac . . . . . 222
Fig. 2. Appearance after Operation . . . . . . . 222
Laryngeal Tuberculosis. Horace Clark, M. D. Fig. View of a Typical Case . . . . . 276
Radical Cure of Inguinal Hernia. S. E. Milliken, M. D. Fig. 1. Dissection of Sac and Exposure of Cord . . . 147
Fig. 2 Suturing of Conjoined Tendon and Poupart's Ligament . . . . . . . . . 148
Fig. 3. Suturing of Flaps of External Oblique . . . . . . 149
Tricuspid Insufficiency. Frank J. Thornbury, M. D. Fig. Anadicrotic Wave Synchronous with Auricular Systole . . . . . . . . . . . . 280

Immigration, Report on, by Dr. Charles S. Hoyt . . . . . . . 689
Immunity, Artificial . . . . . . . 527
Inaugural Address of Dr. Seneca D. Powell . . . . . . . . . . . 435
Infant, Double Synchronous Amputation of Legs in . . . . . . . . 74
Infectious Diseases, Statistics of . 197
Diseases, Causes and Modes of Communication of Contagious and . . . . . . . 201
Influenza or LaGrippe . . . . . . 336
Information, Bits of . . . . . . . 379
Ingraham, Henry D., M. D. Uterine Fibroids, Some Facts in Regard to . . . . . . . . . . . 75
Intestinal Catarrh, Acute . . . . . 338
Indigestion . . . . . . . . . 431
Paralysis—Keen . . . . . 90
Intrauterine Asphyxia, Report of Three Cases . . . . . . . . . . . 65
Intubation . . . . . . . . . . . . 137
Iritis, Croupous . . . . . . . . . 346

PAGE.

JOURNALS, Relation of Medical to the Profession . . . 551
Judson, A. B, M. D. Treatment of Potts' Disease of Spine . . . 143

KERATITIS, Pathology of Hypopyon . . . . . . . . . . . 345
Kidney, Cancer of . . . . . . . 339
Kindergartens, Free . . . . . . . 491
King, Clarence, M. D. Cases from Medical Service at the Cattaraugus County Almshouse . . . 336
Kingsbury, Mrs. Carlton A. Translation of "Lysol" by . . . . 37
Krauss, William C., M. D. Life and Work of Charcot . . . . . 582
What the Newer Therapeutic Procedures Have Done for Neurology . . . . . . . . 212

LAPARATOMY, Exploratory . 19
What Kills Patients After? . . . . . . . . 272
Library of the Surgeon-General's Office . . . . . . . . . . . . . 743
Life Insurance, Suggestions on . . 609

**Literary Notes:**

Alden's Nutshell Cyclopedia . 575
All Around the Year Calendar . 446
American Text-Book of Gynecology . . . . . . . . . . . 319
American Text-Book of Practice . . . . . . . . . . . . 766
American Gynecological Journal . . . . . . . . . . . . 574
Angier Chemical Co. . . . . . 574
An Oldtime Medical Work . . 766
Archives of Pediatrics . . . . 765
A System of Legal Medicine . 447
Balch's Manual for Boards of Health . . . . . . . . . . . 189
Biography of Eminent American Physicians and Surgeons 768
Bristol Medico-Chirurgical Journal . . . . . . . . . . 767
Bulletin of the Medical Society of the Woman's Medical College of Baltimore . . . . 704
Columbian Calendar for 1894 . 380
Cosmopolitan Magazine . . . . . . . . . . 61, 188, 382
Duane's Medical Dictionary . . 190
Dunglison's New Medical Dictionary . . . . . . . . . . 191
Eleanor Fenton . . . . . . . 192
Epitome of Medicine . . . . . 446

PAGE.
Frederick Stearns & Co.'s Calendar . . . . . . . . . . . . 512
Gould's New Illustrated Dictionary . . . . . . . . . . . 189
Gray's Anatomy . . . . . . . 191
Hand-Book of the Board of Regents . . . . . . . . . . . 380
Hernia, Treatment of by Manley . . . . . . . . . . . . 61
Hewson's Anomaly Blanks . . 574
International Medical Annual . 382
International Medical Magazine . . . . . . . . . . . . . 575
Journalistic Changes . . . . . 380
Kansas Medical Journal . . . . 766
Keil's Medical and Dental Directory . . . . . . 511
Ladies' Home Journal . . . . 768
Labordine Chemical Co. . . . 575
Listol Chemical Co . . . . . . 640
Literary Digest, Easter Number . . . . . . . . . . . . . 574
Louisville Medical Monthly . . 575
Madden's Diseases Peculiar to Women . . . . . . . . . . 192
Mark Twain's Romance of an Esquimau Maiden . . . 319
Maryland Medical Journal . . 768
Mathews's Medical Quarterly . . . . . . . . . . . . . . 383, 447
McArthur Hypophosphite Co.'s Calendar . . . . . . . 511
Medical Directory of Connecticut . . . . . . . . . 192
Medical Mirror . . . . . . . 766
Merritt H. Cash Prize Fund . . 765
Musical Contest . . . . . . . 382
National Dispensatory . . 447
New York Engraving & Printing Co.'s Calendar . . . . . 446
New York State Medical Reporter . . . . . . . . . . . 639
Novel Literary Enterprise . . 384
O. W. Clark & Son's Catalogue 640
Pepper's Theory and Practice of Medicine . . . . . . . . 381
Physical Education . . . . . . 61
Piersol's, Normal Histology . . 191
Plimpton Manufacturing Co. . 640
Publication of Transactions of the Pan-American Medical Congress . . . . . . . . . 704
Railway Surgeon . . . . . . . 766
Refractionist, The . . . . . . 640
Rocky Mountain Globe . . . . 767
Samuel D. Gross Prize . . . . 383
Saunders' New Aid Series of Manuals for Students and Practitioners . . . . . . . . 703

PAGE.
Shadow Test . . . . . . . . . 576
Stockton's, The Lady or the Tiger . . . . . . . . . 192
Sousa's Manhattan Beach March . . . . . . . . . . . 320
System of Medical Jurisprudence and Toxicology, by Witthaus and Becker . . 256, 511
Taylor Bros.' Co. . . . . . 640
Teratologia . . . . . . . . . . 767
Transactions of American Association of Obstetricians and Gynecologists, Vol. VI. . 446
Uncle Tom's Cabin . . . . . 62
Vandegrift's Digest of the Tariff Bill . . . . . . . . 765
Vick's Floral Guide . . . . . 576
Visiting List, Antikamnia Chemical Co.'s . . . . . . . 511
William R. Warner & Co., Award of Silver Medal to . . 704
Woman's Medical College of Baltimore . . . . . . . . . 575
Woman's Medical Journal . . 511
Young Man in Business . . . 576
Long, J. W., M. D. The Value of Hegar's Sign of Pregnancy 706
Benj. G., M. D. Puerperal Convulsions with Albuminuria . . . . . . . . . . . 723
Love, I. N., M. D. Neurasthenia from Standpoint of General Practitioner . . . . . . . . . . . 513
Lunacy Commission, State . . . . 490
Lysol (Translation) . . . . . . . . 37

MALTINE Manufacturing Co.'s Calendar . . . . 339, 433
Manley, Thomas H., M. D. Torsion and the Homologous Ligation of Divided Arteries . . . . 257
Mann, Matthew D., M.D. Exploratory Laparatomy . . . . . . . . 19
Massachusetts Institute of Technology . . . . . . . . . . . . 226
Massage for Sprains and other Joint Injuries—Lee . . . . . . . . . 97

**Medical College Notes:**

Annual Announcements . . . . 117
Buffalo University . . . . . . 117
Election of Class Officers . 365
Faculty Changes . . . 117, 627
Hospital College of Medicine, Louisville . . . . . . . . 432
Jefferson Medical College . . . 437
Medico-Chirurgical College of Philadelphia . . . . . . . . 751

PAGE.

Niagara University . . . 117, 628
Northwestern University, Chicago . . . . . . . 751
Post-Graduate Medical School and Hospital . . . . . . . 688
Rush Medical College . . . . 437

Medical Education, Efforts of Medical Society of the State of New York in Advancing . 170
Libraries, Presentations of . 298
Meniere's Disease, Salicylate of Sodium in . . . . . . . . . . 156
Midwife, Death Through Neglect of, 550
Midwives, Proposed Bill to License, 357
Migraine . . . . . . . . . . . 136
Milliken, Samuel E., M. D. Review of Operative Treatment for Radical Cure of Inguinal Hernia . . . . . . . . . . . 146
Montana Board of Medical Examiners *vs.* Dr. E. S. Kellogg . . 619
Mynter, Herman, M. D. Clinical Memoranda from the Sisters' of Charity Hospital . . . . . . . .
. . 221, 281, 341, 415, 532, 588, 718
Myoma, Suppurating, of Uterine Wall, Followed by Twin Pregnancy . . . . . . . . . . . . . 542
Myxedema . . . . . . . . . . 155

NASAL Passages, Cysts of . . . 150
Neisser, Professor A., on the Microscope in Examinations for Gonorrhea . . . 471
Nephrolithiasis, Action of Glycerine in . . . . . . . . . . . . . 110
Neurasthenia, From Standpoint of General Practitioner . . . . . . 513
Neuritis, Senile Form of Multiple, 155
Neurology, What the Newer Therapeutic Procedures Have Done for . . . . . . . . . . . . . . 212
Notes of the Pan-American Congress . . . . . . . . . . . . . . 168
Nurses, Male . . . . . . . . . . . 488
Points for Trained . . . . . 531

### Obituaries:

Billroth, Dr. Theodor . . . . 494
Brown-Sequard, Charles E., M. D. . . . . . . . . . . . . . 624
Clark, Sir Andrew, Bart. . . . 302
Dayton, Charles L., M. D. . . 172
Dunlap, Alexander, M D. . . . 555
Earle. Charles Warrington, M. D. . . . . . . . . . . . . 301

PAGE.

Edis, Arthur Wellesley, M. D. 366
Elder, Elijah, S., M. D . . . 746
Ford, Corydon L., M. D. . . . 625
Hewitt, Graily, M. D. . . . 171
Hill, Hampton Eugene, M. D. 555
Judd, Herbert, M. D. . . . . 437
Keating, John M., M.D., LL.D. 302
Loop, Mrs. Rebecca . . . . . 625
Maisch, John M., M. D . . . . 236
Mallory, M. L., M. D. . . . 693
Marselius, Willard C., M. D. . 436
McCann, James, M. D., LL. D. 50
Nesbitt, Geo. W., M. D. . . . 693
Rauch, John H., M. D. . . . . 624
Stevens, A. A., M. D. . . . . 237
Van Aernam, H., M. D . . . . 744
Vought, Walter, M. D. . . 171, 237

OHIO, Medical Examining Board of . . . . . . . . 554
Operating Room, New, at the Sisters' of Charity Hospital . . . 363
Opium Habit in Mother, Effect of, on New-born . . . . . . . . . 157
Ophthalmic Instruments, Sterilization of . . . . . . . . . . . 64
Orleans Parish Medical Society . . 300
Ovary, Hematoma of . . . . . . . 733

PAN-AMERICAN Medical Congress, Tour of Foreign Delegates . . . . . . . . . . . . 168
Page, R. C. M., M. D. Functional Dyspepsia . . . . . . 602
Parmenter, John, M. D. Annual Address of, as President Medical Society of the County of Erie . . . . . . 449
John, M. D., Empyema . . . 321
Paranoia, with Delusions of Change in Sex . . . . . . . . . . . . . 193
Paralysis, Obstetric . . . . . . . 156
Patient, What Kills, Peritonitis or Infection? . . . . . . . . . . 711
Pertussis Treated with Bromoform 108
Perineum, Superficial Laceration of Female . . . . . . . . 157
Pennsylvania Railroad Company, Vaccination of Employees of . . . . . . . . . . . . 589
State Boards of Medical Examiners of . . . . . . . . 489

### Personals:

Abbott, Frank W., M. D. . . . 48
Brown, Ira C., M. D. . . . . . 691
Brown, M M., M. D. . . . . . 554
Buswell, Henry C., M. D. . . 764

PAGE.

Campbell, Albert L. D., M. D. 692
Carroll, Jane W., M. D. . . . . 748
Carstens, J. Henry, M. D. . . . 493
Clark, Horace, M. D. . . . . . 365
Coggin, William T., M. D. . . 492
Cott, George F., M. D. . . . . 301
Crockett, M. A., M. D. . . . . 493
Cronyn, John, M. D. . . . 49, 749
Currier, Andrew F., M. D. . . 692
Daniels, C M., M. D. . . . . 554
Dean, H. J., M. D. . . . . . . 116
Dorr, L. Bradley, M. D. . . . 49
Dooley, E. M, M. D. . . . . . 49
Ewing, A. M., M. D. . . . . . 692
Finerty, J. J., M. D. . . . . . 693
Flagg, John D., M. D. . . 48, 692
Frost, Edward L., M. D. . . . 691
Frye, Maud J., M. D. . . . 49, 554
Grant, H. Y., M. D. . . . . 692
Grove, B. H., M. D. . . 378, 748
Hamilton, John B., M. D. . . . 49
Hart, Ernest, M. D. . . . . 64, 114
Harrington, F. A, M. D. . . . 691
Hayes, F. A., M. D. . . . . . 49
Hewitt, J. W., M. D. . . . . . 692
Hinkel, F. W., M. D. . . . . . 692
Holmes, J. B. S, M. D. . . . 624
Howe, Lucien, M. D. . . . . . 436
Hooper, P. O., M. D. . . . . . 493
Hubbell, A. A., M. D. . . . . 49
Hulbert, George F., M. D. . . 624
Hummel, A. L., M D. . . . . 748
Hummel and Parmele . . . 49, 379
Jacobi, Abraham, M. D. . . . 364
Krauss, Wm. C., M. D. . . . 171
Lanphear, Emory, M. D. . . . 747
Leonhardt, H. C., M. D. . . . 171
Long, C. E., M. D. . . . . . . 691
Long, J. W., M. D. . . . . . . 365
Lothrop, Thomas, M. D. . 49, 748
Macbeth, A. H., M. D. . . . . 669
Manges, Monroe, M. D. . . . . 911
McMurtry, L. S., M. D. . . . . 748
Mead, Harry, M. D. . . . . . 691
Miller, A. B., M. D. . . . . . 301
Morrison, D. A., M D. . . . 749
Mulford, Henry J., M. D. . . . 301
Murdock, J. B., M. D. . . . . 747
Murphy, J. B., M. D. . . . . . 691
Myers, W. H., M. D. . . . . . 435
Norris, Richard C., M. D. . . 435
Owen, A. M., M. D. . . . . . 493
Park, Roswell, M. D. . . 236, 365
Parmenter, John, M. D. . . 48
Peterson, Frederick, M. D. . . 49
Pepper, William, M. D. . . . . 628
Pettit, John A., M. D. . . . . 691
Preiss, Frederick, M. D. . . . 49
Price, Joseph, M. D. . . . . . 435

PAGE.

Pohlman, Julius, M. D. . . . . 747
Porter, Miles F., M. D. . . . . 435
Powell, Seneca D., M. D. . . 365
Redmond, D. L., M. D. . . . 49
Reed, Charles A. L, M. D. . . 748
Reed, R. Harvey, M. D. . . . 493
Richmond, Nelson G., M. D. . . . . . . . . . . . . 116, 692
Rohé, George H., M. D. . . 691
Siegfried, C. S., M. D. . . . . 554
Smith, Eugene, M. D. . . . . 436
Stockton, Charles G., M. D. . . 748
Suiter, A. Walter, M. D . . . 171
Taylor, Wm. G., M. D. . 692, 49
Tremaine, W. S, M. D. . . 692
Vadeboncoeur, A. F., M. D . 748
Walker, Edwin, M. D. . . . . . 493
Wende, Ernest, M. D. . . . . 365
Wetmore, S. W.. M. D. . . . . 748
Mary Berkes, M. D. . . . 748
Winn, J. F., M. D. . . . . . . 554
Wood, Harry A., M. D. . . . 48

Physicians' (N. Y.) Mutual Aid Association, Report of . . . 621
Personality and Its Influence 577
Post-office Building in Buffalo, Unsanitary Condition of . . . . . 115
Post-Operative Sequelæ of Pelvic and Abdominal Surgery—Price . 95
Posture, Concerning . . . . . . 85
Erect for Gynecological Examinations . . . . . . . . 299
Potter, William Warren, M. D. Puerperal Sepsis, etc. . . 10
William Warren, M. D. The Prevention of Disease, a Problem for all Physicians 647
Polyclinics in Berlin . . . . . . . 105
Potts' Disease of the Spine, Treatment of . . . . . . . . . . . . 143
Potassium, Permanganate, an Antidote for Morphine . . . . . . . 456
Politics, Medical in Medical Societies . . . . . . . . . . . . . . 490
Pregnancy, The Value of Hegar's Sign of . . . . . . . . . . . . 706

**Progress in Medical Science:**

Nervous Diseases. By James W. Putnam, M. D. . . . . . . 154
Ophthalmology and Otology. By A. A. Hubbell, M. D. . . 345
Surgery. By John Parmenter, M. D. . . . . . . . . . . . . 89

Prostate, White's Operation for Enlarged . . . . . . . . . . 481
Prostatic Diseases of Old Age . . . 397

PAGE.

Price, Joseph, M. D. Disputed Points in Hysterectomy . . . . . 26
Prize. The Samuel D. Gross . . . 383
The William F. Jenks . . . 126
The Merritt H. Cash . . . . 765
Public Instruction, Report of Superintendent of for New York for 1893 . . . . . . . . . . . . . . . 623
Puerperal Convulsions with Albuminuria . . . . . . . 723
Sepsis: Its Prevention and Cure . . . . . . . . . . 10

QUARANTINE, Maritime . . . 659

RECTAL AFFECTIONS in Young Children . . . . . . 612
Rectum, Extirpation by the Kraske Method . . . . . . . . . . . 732
Refraction, Errors of Epileptics . . 347
Regents, Diploma of . . . . . . . 544
Of the University . . . . 743
Reed, Charles A. L. Reed, M. D. Secretary-General, etc., Presentation to . . . . . . . . . . . 169
Republic, Grand Army of . . . . . 235
Renner, W. Scott, M. D. Cysts of Nasal Passages . . . . . . . . 150

**Reviews:**

Alt: Ophthalmology for the General Practitioner . . . . 375
Anderson: Mineral Springs and Health Resorts of California . . . . . . . . . . . 508
Ashurst: Principles and Practice of Surgery . . . . . . 503
Balch: Manual for Boards of Health . . . . . . . . . 507
Baldy: American Text-Book of Gynecology . . . . . . . 559
Ballantine: Structures of the Mesosalpinx . . . . . . . . . 123
Bartholow: Cholera . . . . . 314
Beard: Treatise on Nervous Exhaustion . . . . . . . . . 636
Bouchard: Lectures on Auto-Intoxication in Disease . . . 696
Browne: Diseases of the Throat and Nose . . . . . . 372
Bratenahl and Tousey: Gynecology . . . . . . . . . . . . 125
Brockway: Essentials of Physics . . . . . . . . . . . . . 762
Buck: Supplement to the Reference Hand-Book of the Medical Sciences . . . . . . . 307

PAGE.

Burr: Primer of Psychology and Mental Disease . . . . 632
Bushong: Modern Gynecology 305
Carmichael: Diseases of Children. . . . . . . . . . . . . 59
Cerna: Notes of the Newer Remedies . . . . . . . . . . 251
Chapman: Manual of Jurisprudence and Toxicology . . 314
Chetwood: Genito-Urinary and Venereal Diseases . . . 124
Coplin and Bevan: Manual of Practical Hygiene . . . . . 758
Currier: Art of Preserving Health . . . . . . . . . . . 241
Davis: Diseases of the Heart, Lungs and Kidneys . . . . . 186
Delafield: Hand-Book of Pathological Anatomy and Histology . . . . . . . . . 56
Doubleday: Practice of Medicine . . . . . . . . . . . 375
Duane: Students' Medical Dictionary . . . . . . . . . 369
Dunglison: Dictionary of Medical Science . . . . . . 311
Era Key to the United States Pharmacopeia . . . . . . . 374
Ewart: Cardiac Outlines . . 183
Field: Diseases of the Ear . . 373
Firebaugh: The Physician's Wife . . . . . . . . . . . 699
Flückiger: Reactions . . . 309
Foster: Encyclopedic Medical Dictionary . . . . . . . 557
Gage: Microscope and Microscopical Methods . . . . . . 637
Gallaudet: Surgery . . . . . 374
Garrigues: Text-Book of Diseases of Women . . . . . . 755
Gowers: Diseases of the Nervous System . . . . . . . . 565
Graham: Recent Developments in Massage . . . . . 310
Gray: Descriptive and Surgical Anatomy . . . . . . . 370
Hammarsten: Text-Book of Physiological Chemistry . . 504
Harte: Hand-Book of Local Therapeutics . . . . . . . . 58
Heath: Minor Surgery and Bandaging . . . . . . . . . 702
Heron: Evidences of Communicability of Consumption 245
Hibbard: Diet for the Sick . 55
Hirt: Diseases of the Nervous System . . . . . . . . . 242
Holden, W. A.; Embryology of the Eye . . . . . . . . 567

PAGE.

Hyde: Treatise on Diseases of the Skin . . . . . . . . 502
Index-Catalogue, Library Surgeon-General's Office, U. S. Army . . . . . . . . . . . . 311
International Medical Annual and Practitioner's Index . . . 631
Jackson: Treatise of Diseases of the Hair and Scalp . . . 636
Jacobi: Clinical Lectures on Pediatrics . . . . . . . . . 761
Johns Hopkins Hospital Report in Gynecology, 698; Volume III., Pathology . . . 759
Juler: Hand-Book of Ophthalmic Science and Practice . . . . . . . . . . . 371
Keating: International Clinics. Vol. I., 3d Series, p. 57; Vol. II., 3d Series, p. 568; Vol. III., 3d Series, p. 500; Vol. IV., 3d Series 757
Keen: Operation Blanks . . 317
Kraft-Ebing: Psychopathia Sexualis . . . . . . . . . . 249
Kocher: Operative Surgery . 700
Langton: Holden's Anatomy 634
Landis: How to Use the Forceps . . . . . . . . . . . . . 633
Leonard: Physicians' Pocket Day-Book . . . . . . . 312
Loomis: Lessons in Physical Diagnosis . . . . . . . . . 183
MacDonald: Abnormal Man (Bureau of Education) . . 508
Maisch: Manual of Organic Materia Medica . . . . . . . 247
Manley: Hernia . . . . . . . 442
Manning: Physiology . . . . 375
Manuel de Medecin Practicien 762
Marcy: Anatomy and Surgical Treatment of Hernia . . 54
Martin: Essentials of Minor Surgery . . . . . . . . . . 507
Mathieu: Diseases of Stomach and Intestines . . . . . . 528
McLaughlin: Fermentation, Infection and Immunity . . 185
McMurtry: Nursing in Pelvic Surgery . . . . . . . . 698
McNutt: Diseases of the Kidneys and Bladder . . . 182
Miller: Diseases of the Eye, Ear, Throat and Nose . . . 125
Minot: Human Embryology . 179
Morrow: System of Genito-Urinary Diseases, Syphiology and Dermatology. Vol. II. . . . . . . . . . . . . . 494
Morrow: Venereal Memoranda . . . . . . . . . . . . . 701
Musser: Treatise on Medical Diagnosis . . . . . . . . . 695
Napheys: Modern Therapeutics . . . . . . . . . . . . 184
Norris: Text Book of Ophthalmology . . . . . . . . 303
Syllabus of Obstetrical Lectures . . . . . . . . . . 759
Parkes: Hygiene and Public Health . . . . . . . . . . . 181
Pellew: Manual of Practical, Medical and Physiological Chemistry . . . . . . . . . . 253
Pepper: Text-Book of Theory and Practice of Medicine, Vol. I., 177; Vol. II. . . . . 569
Piersol: Text-Book of Normal Histology . . . . . . . . 630
Pharmacopeia of the United States . . . . . . . . . . . . 501
Playfair: Treatise on Science and Practice of Midwifery . 497
Pye-Smith: Diseases of the Skin . . . . . . . . . . . . . 375
Remondino: Treatment of Pulmonary Consumption in Southern California . . . . 637
Robinson: Electro-Therapeutics of Neurasthenia . . . 310
Roe: Foreign Bodies in the Larynx and Trachea . . . . 562
Sajous: Annual of Universal Medical Sciences . . . . . 248
Sanitary Reports of Marine Hospital Service . . . . . . 370
Savage: New Truths in Ophthalmology . . . . . . . . 443
Schæffer: Essentials of Histology . . . . . . . . . 316
Schæffer: Pocket Atlas of Obstetrics . . . . . . . . . . 252
Schofield: Elementary Physiology for Students . . . . . 250
Senn: Syllabus of Lectures on Surgery . . . . . . . . . . . 700
Simon: Manual of Chemistry 186
Starr: American Text-Book of Diseases of Children . . . . 752
State Board of Charities, Annual Report of . . . . . . . 558
Struthers: Chemistry and Physics . . . . . . . . . . . 563
State Board of Health of New York, Twelfth and Thirteenth Annual Reports . . . 571
Starling: Elements of Human Physiology . . . . . . . . . 250

PAGE.

Sternberg: Manual of Bacteriology . . . . . . . . . . 244
Stevens: Manual of the Practice of Medicine . . . . . 253
Manual of Therapeutics . . 761
Stillé: National Dispensatory 505
Stirling: Outlines of Practical Histology . . . . . . . . 309
Stockwell: Cholera . . . . . 310
Tanner: Memoranda of Poisons . . . . . . . . . . . . 316
Thorpe: Benjamin Franklin and the University of Pennsylvania . . . . . . . . . . 239
Transactions American Association of Obstetricians and Gynecologists, Vol. V., 249; Vol. VI. . . . 634
American Gynecological Society, Vol. XVII., 317; Vol. XVIII. . . . . . . 563
American Laryngological Association for 1892 . . 374
American Surgical Association, Vol XI. . . . . . . 501
Association of American Physicians, Vol. VIII. . 316
Medical Society of the State of New York . . 247
Medical Society of the State of Kentucky . . . 251
Medical and Chirurgical Faculty of the State of Maryland, 1890-'91, 312; 1892-'93 . . . . . . . . . 570
Medical Society of the State of North Carolina . . . . (1892) 315, (1893) 760
Medical Association of Georgia . . . . . . . . . 506
Philadelphia County Medical Society. Vol. XIII., 315; Vol. XIV. . . . . 701
Southern Surgical and Gynecological Association, Vol. V., 313; Vol. VI. . . 760
Texas State Medical Association, 1892 and 1893 . 313
Trouessant: Antiseptic Therapeutics . . . . . . . . . . 637
Tyson: Physical Diagnosis . 570
Van Valzah: Chronic Disorders of the Digestive Tube . 254
Visiting List, Blakiston's for 1894 . . . . . . . . . . . 371
Visiting List, the Medical News of 1894 . . . . . . . . . . . 308
Vought: Chapter on Cholera . . . . . . . . . . . . . 252

PAGE.

Warner: Materia Medica and Therapeutics. . . . . . . . 254
Wharton: Minor Surgery and Bandaging . . . . . . . . . 368
Witthaus and Becker: Medical Jurisprudence, Forensic Medicine and Toxicology . . 754
Year-Book of Treatment for 1893. . . . . . . . . . . 60
Year-Book of Treatment for 1894 . . . . . . . . . . . 635
Yeo: Manual of Medical Treatment . . . . . . . . . 368
Rochester, De Lancey, M. D. Prevention of Disease . . . . . . . . 1
Robinson, F. Byron, M. D. What Kills Patients after Laparatomy—Anesthetic, Nephritis or Infection? . . . . 272
What Kills the Patient, Peritonitis or Infection? . . . 711
Rohé, George H., M. D., Hematoma of the Ovary . . . . . . . . . 733
Ross, James F. W., M. D. Suppurating Myoma of Uterine Wall . 542

SANITARY Inspection by Buffalo Health Department . . . 617
Sanitary Negligence, Cost of . . . 549
Scarlet Fever, How Shall we Treat? 78
Treatment of . . . . . . . 608
School Books, Errors in . . . . . . 47
Life and Physical Development . . . . . . . . . . . 129
Schoolhouses, Three-storied and General Field . . . . . . . . . 362
Senn, Nicholas, M. D., Gift of to Newberry Public Library . . 486
Sewerage System, the New Orleans 741
Shrady, Dr. George F., and the Marion Sims' Statue . . . . . . . . 689
Sims, Dr. J. Marion, Statue of . . 689
Smallpox in Chicago . . . . . . . 689

**Society Meetings:**

American Academy of Medicine . . . . . . . . . . . . 749
American Association of Obstetricians and Gynecologists 694
American Electro-Therapeutic Association . . . . . . . 53, 302
American Dermatological Association . . . . . . . . 121, 627
American Medical Association, Section on Surgery and Anatomy . . . . . . . . . . . 556
American Physicians and Surgeons, Congress of . . . . . 557

PAGE.
American Medical Publishers' Association . . . . . . 378, 625
American Social Science Association . . . . . . . . . 122
American Surgical Association 626
Association of Erie Railway Surgeons . . . . . 366, 693, 764
Association of Military Surgeons of U. S. . . . . . . 557, 627
Association of Western New York and Pennsylvania Railway Surgeons . . . . . . 626
Cleveland Medical Society . . 627
International Congress of Obstetrics and Gynecology . . 749
International Medical Congress, Eleventh . . . 116, 303, 367, 438
Medical Association of Central New York . . . . . . . . . 49
Medical Association of Georgia . . . . . . . . . . . . 557
Medical Society of the County of Cattaraugus . . . . . . 749
Medical Society of the County of Chautauqua . . . . . 367, 749
Medical Society of the County of Erie . . . . . . . . . . 694
Medical Society of the State of New York . . . . 176, 366, 439
Medical Society of the State of Pennsylvania . . . . . . . . . 553
Michigan State Medical Society 697
Mississippi Valley Medical Association . . . . . . . . . . 173
National Association of Railway Surgeons . . . . . . . 627
New York State Association of Railway Surgeons . . . . . 238
Ohio State Medical Society . . 176
Pan-American Medical Congress . . . . . . . . . . . 115
Pan-American Medical Congress. Committee of Arrangements . . . . . . . . . 128
Pan-American Medical Congress, Excursion to Rome . . 62
Pan-American Medical Congress, Official Delegates to . 63
Pan-American Medical Congress, Program, Section on Gynecology and Abdominal Surgery . . . . . . . . . . 118
Pan-American Medical Congress, Section on Materia Medica and Pharmacology . 63
Pan-American Medical Congress, Section on Hygiene, Climatology and Demography . . . . . . . . . . . . 123

PAGE.
Pan-American Medical Congress, Section on Diseases of Children . . . . . . . . . 127
Roswell Park Medical Club . . 626
Southern Surgical and Gynecological Association . . . . . 238
Tri-State Medical Society . . . 302

**Society Proceedings**:
Buffalo Academy of Medicine . 590
Medical Society of the County Erie . . . . . . . . 22, 420, 725
Philadelphia County Medical Society . . . . . . . . . . 26

Society Transactions and Libraries 297
Southern Pines, Winter Health Resort . . . . . . . . . . . . . 297
Spelling of Some Medical Words . . 42
Spinal Concussion . . . . . . . . 107
Stapes, Removal of . . . . . . . . 349
State Examining Fees, Reduction of . . . . . . . . . . . . . . . 487
Stenography, Medical . . . . . . . 486
Street Cars, Cold on Elmwood Avenue . . . . . . . . . 360
Heating of . . . . . . . . . 298
Students' Fees for Tuition vs. State Examination . . . . . . . . . . 234
Student, the Smart Medical and the State Examining Fees . . . . . 486
Substitution, Evils of . . . . . . . 287
Surgeons, Acting Assistant Petitioning Congress . . . . . . . . . . 553
Surgical Suggestions . . . . . . . 377
Operations, Liability for Injuries when Death follows 612
Problems in Intra-pelvic and Abdominal Diseases . . . 727
Syphilitics, Marriage of . . . . . 713

TERRALINE, Experiments with 340
Tibia, Tuberculous Epiphysitis of . . . . . . . . . . 282
Thermometer, An Accurate Clinical 619
Thornbury, F. J., M. D. Tricuspid Insufficiency . . . . . . . . . 278
Thompson, J. C., M. D. Physicians' Personality and its Influence . . . . . . . . . . . . 577
Topics of the Month . 47, 114, 231, 296, 357, 432, 486, 549, 617, 687, 741
Torticollis, Spasmodic . . . . . . 154
Tricuspid Insufficiency . . . . . . 278
Trional, Indications for . . . . . . 290
Trephining, Twenty-seven Cases of . . . . . . . . . . . . . . . 532

PAGE.

Tuberculosis, Report of Committee on, to Buffalo Academy of Medicine . . . . . . . . . 590
Laryngeal, A Typical Case of . . . . . . . . . . . . 275
in Relation to Animal Industry and Public Health . . 621
Circular of New York City Health Department, Relating to . . . . . . . . . . 741
Turkish Baths . . . . . . . . . 233
Typhoid Fever, Epidemic at Montclair, N. J. . . . . . . . . . 620
Statistics in Buffalo for March, 1894 . . . . . . . 622
Cause of in General and of the Epidemic in Buffalo (in March) in Particular . . . 641
Prevention of . . . . . . . 641

URIC ACID, Anemia and Blood Changes Caused by . . . . 158
Urinary Incontinence in Woman, Surgical Treatment of . . . . . 291
Uterine Appendages, Results of Removal of Diseased . . . 385
Fibroids, Treatment of . . . 482

PAGE.

Uterine Fibroids . . . . . . . . . . 75
Uterus, Pregnant—Exploratory Laparatomy . . . . . . . . . . . 19

VERTEBRA, Dislocation of Cervical . . . . . . . . . . 159
Partial Dislocation of Cervical . . . . . . . . . . 339

WARREN-BEY, Edward, M. D., Anecdote of . . . . 359
Water and Health Departments . . 551
Weigel, Louis A., M. D., School Life and Physical Development . 129
Werder, X. O., M. D., Extirpation of the Rectum by the Kraske Method . . . . . . . . . . . . . 732
Wheeler, William A., M. D., Maritime Quarantine . . . . . . . . . 659
Whipple, Electa B., M. D., Dysmenorrhea . . . . . . . . . . . 457
Women, Gonorrheal Infection in . 654
Students in Paris . . . . . . 84
Women's Medical Club Banquet . . 687
Words, Spelling of Some Medical . 42

